# Building deep learning and traditional chemometric models based on Fourier transform mid‐infrared spectroscopy: Identification of wild and cultivated *Gastrodia elata*

**DOI:** 10.1002/fsn3.3565

**Published:** 2023-07-11

**Authors:** Shuai Liu, Honggao Liu, Jieqing Li, Yuanzhong Wang

**Affiliations:** ^1^ College of Agronomy and Biotechnology Yunnan Agricultural University Kunming China; ^2^ Medicinal Plants Research Institute Yunnan Academy of Agricultural Sciences Kunming China; ^3^ Yunnan Key Laboratory of Gastrodia and Fungi Symbiotic Biology Zhaotong University Zhaotong China

**Keywords:** authentication, chemometrics, deep learning, Fourier transform mid‐infrared (FT‐MIR) spectroscopy, *Gastrodia elata*, three‐dimensional correlated spectral (3DCOS)

## Abstract

To identify wild and cultivated *Gastrodia elata* quickly and accurately, this study is the first to apply three‐dimensional correlation spectroscopy (3DCOS) images combined with deep learning models to the identification of *G. elata*. The spectral data used for model building do not require any preprocessing, and the spectral data are converted into three‐dimensional spectral images for model building. For large sample studies, the time cost is minimized. In addition, a partial least squares discriminant analysis (PLS‐DA) model and a support vector machine (SVM) model are built for comparison with the deep learning model. The overall effect of the deep learning model is significantly better than that of the traditional chemometric models. The results show that the model achieves 100% accuracy in the training set, test set, and external validation set of the model built after 46 iterations without preprocessing the original spectral data. The sensitivity, specificity, and the effectiveness of the model are all 1. The results concluded that the deep learning model is more effective than the traditional chemometric model and has greater potential for application in the identification of wild and cultivated *G. elata*.

## Graphical abstract

To identify wild and cultivated *Gastrodia elata* quickly and accurately, this study is the first to apply three‐dimensional correlation spectroscopy (3DCOS) images combined with deep learning models to the identification of *G. elata*. The results concluded that the deep learning model is more effective than the traditional chemometric model and has greater potential for application in the identification of wild and cultivated *G. elata*.

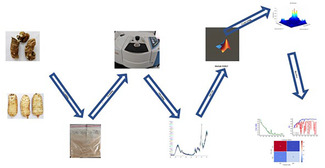

## 1 INTRODUCTION

*Gastrodia elata* is a genus of plants in the orchid family Gastrodia, whose earliest use in Chinese history was recorded a thousand years ago. The dried and steamed root is used as a folk medicine and is known as “TianMa” (Zhao et al., 1999). It improves nervousness, relieves insomnia and headaches, and has sedative, anti‐inflammatory, and sleeping effects (Qiu et al., 2014; Wang, Chen, & Li, 2014; Wang, Zhang, et al., 2014). The shortage of wild *G. elata* resources has led to the rapid development of the cultivated *G. elata* industry. Cultivated *G. elata* occupies a large proportion of the market and has become the main source of *G. elata*. However, in terms of medicinal value, there are differences between wild *G. elata* and cultivated *G. elata* (Pei et al., 2022). The shortage of wild resources and the difference in medicinal value led to a higher value of wild *G. elata* than cultivated ones, which allows unscrupulous traders to see huge profits (Chen et al., 2021). It is difficult to identify cultivated and wild *G. elata* by the naked eye, and the traditional way of identification is by morphology, which is more subjective and difficult to ensure accuracy by empiricism (Liu, 2012). And for inexperienced consumers, it is difficult to distinguish between wild and cultivated *G. elata* differences. Illegal businessmen take advantage of this to package the cultivated *G. elata* as wild *G. elata* and sell them to consumers for profit. Therefore, the market and consumers urgently need a fast, accurate, and effective technical method to identify wild and cultivated *G. elata* in order to protect the rights and interests of consumers and businesses, and maintain market order.

At present, the main methods for species identification are electronic nose, chromatography, mass spectrometry, spectroscopy, molecular marker technology, and genome sequencing technology (Wei et al., 2022). Spectroscopy is an emerging technology that has gained a lot of recognition in recent years. The benefits are extremely obvious compared to other technical methods. The preparation of experimental samples is simple and has the advantages of being nondestructive, green, efficient, and convenient. The operation of the spectroscopic instrument is simple and does not require a professional to operate; secondly, the process of analyzing the obtained spectral data is not complicated and does not require a lot of time to process the data.

Fourier infrared spectroscopy (FTIR) combined with traditional chemometrics or deep learning to build models, which has shown excellent results in prediction and identification studies of species. However, preprocessing spectral data is essential before building a chemometric model. Problems such as light scattering and baseline instability from the sample itself and the instruments need to be reduced. The accuracy and stability of the model can be improved by selecting the appropriate preprocessing methods to minimize some adverse effects. This is a necessary step for traditional chemometrics combined with spectroscopic data to build a model (Chen et al., 2023; Wang et al., 2022; Zhang et al., 2021). There are a number of difficulties in analyzing one‐dimensional spectra, such as the overlap of spectral information from large sample sizes and the difficulty in obtaining detailed information. In this case, two‐dimensional correlation spectroscopy (2DCOS) images have emerged, which have the advantage of solving the problem of overlapping information in one‐dimensional spectra and better displaying the spectral absorption peaks of the samples. At the same time, 2DCOS can express the intensity relationship of the spectral signal and improve the visibility of the spectral information (Noda, 2018).

Solving the species identification problem by using 2DCOS combined with deep learning models, Yue et al. (2021) directly used the raw spectral data to generate 2DCOS images to build a residual convolutional neural network (ResNet) model with 100% accuracy and concluded that the accuracy of the model would not be improved by using the preprocessing method. ResNet of CNN networks is the most widely used, which has the advantage of eliminating the problem of gradient explosion and disappearance (Dong et al., 2023). Recently, Chen, Liu, et al. (2022) used the 2DCOS‐ResNet model to solve the problem of origin tracing of species of wild porcini mushrooms, and the classification accuracy of the model was satisfactory; Yan et al. (2022) compared the 2DCOS‐ResNet model with traditional chemometrics models and demonstrated that the 2DCOS‐ResNet model outperformed the traditional chemometrics models; Chen, Li, et al. (2022), successfully identified different storage periods of wild porcini mushrooms using 2DCOS‐ResNet with 100% accuracy. Li et al. (2022) proposed a 3DCOS combined with a deep learning model for the species identification problem of wild porcini mushrooms. The advantage of 3DCOS images is that the change from planar to spatial makes the spectral absorption peaks more pronounced, which allows the deep learning model to capture a fuller information of the image.

In this paper, 3DCOS images are combined with deep learning models for the first time to solve the identification problem of wild and cultivated *G. elata*, and PLS‐DA and SVM models are built for comparison with deep learning models. Before building these chemometric models, the raw spectral data were processed by first‐order derivative (1D), 2D, multiplicative scattering correction (MSC), standard normal variate transformation (SNV), and Savitzky–Golay (SG), and their combinations. The above operations were performed to compare the effects of two chemometric models under different preprocessing methods. The 3DCOS‐ResNet model was built on the basis of the raw spectral data, and then the model performance was evaluated and compared with PLS‐DA and SVM models. Through comparison, a most suitable identification model was found for the identification of wild and cultivated *G. elata*, which will provide a valuable technical reference for future practical application in the market.

## 2 MATERIALS AND METHODS

### 2.1 Collection and treatment of samples

**FIGURE 1:**
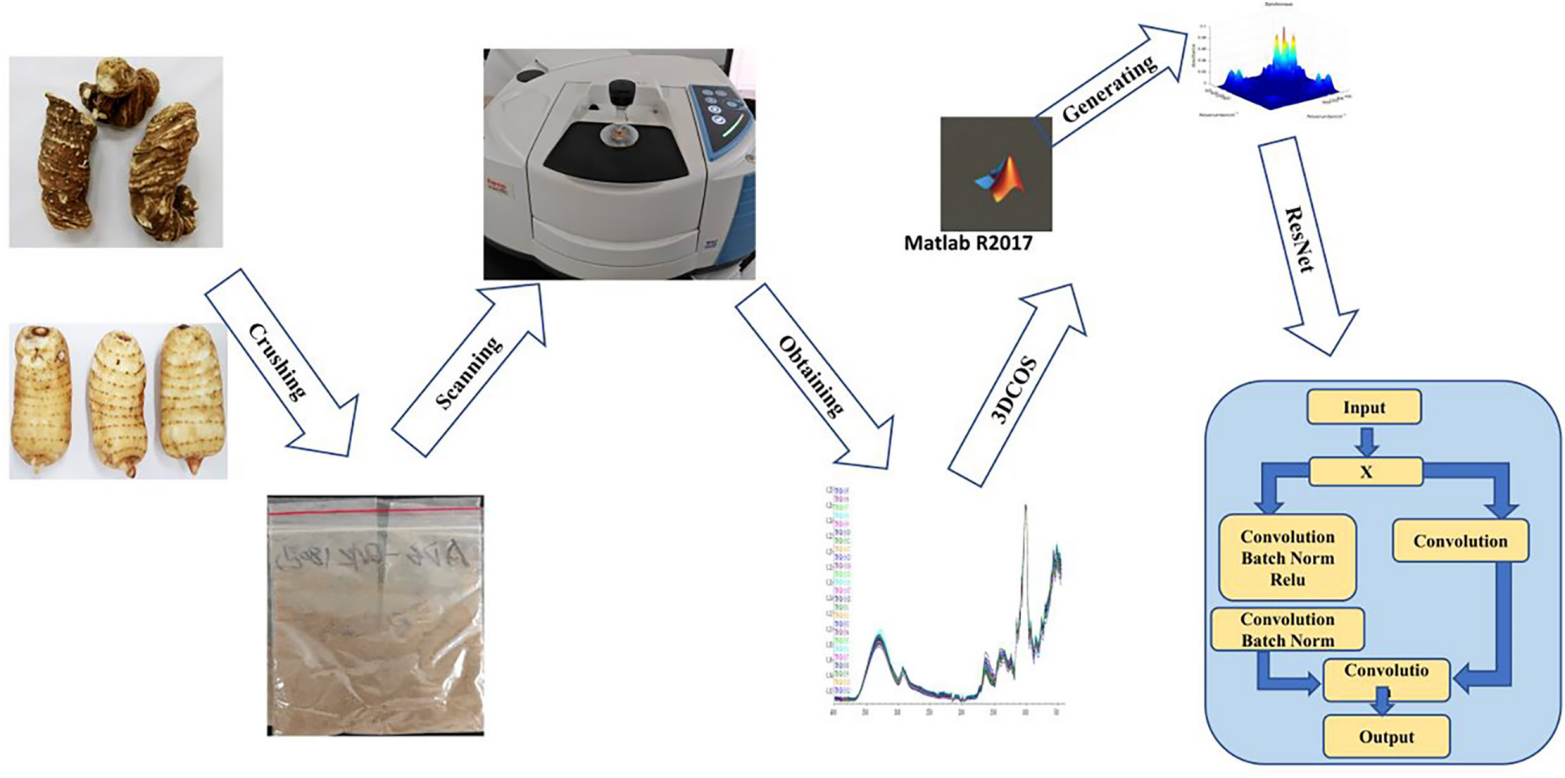
Flow chart of sample preparation and three‐dimensional correlated spectral images combined with residual convolutional neural networks.

All samples of *G. elata* were collected from Yunnan Province of China. There are 172 samples including 109 cultivated and 63 wild *G. elata*. Details of information are shown in Table S1. The samples studied were certified by Wang Yuanzhong, a researcher at the Institute of Medicinal Plants, Yunnan Academy of Agricultural Sciences. After removing the soil and other impurities from the surface of the collected samples, they were rinsed with pure water and dried. All samples were placed in the oven at 60°C until they reach a constant weight and then removed. The samples were made into powder using a DFY‐500 high‐speed crusher (Wenling Linda Machinery Co., Ltd.), filtered through a 100‐mesh stainless steel sieve tray, and then stored in polyethylene bags at room temperature and protected from light. The flow chart for the preparation of the experimental samples is shown in Figure 1.

### 2.2 Acquisition and preprocessing of FT‐MIR spectrum data

**FIGURE 2:**
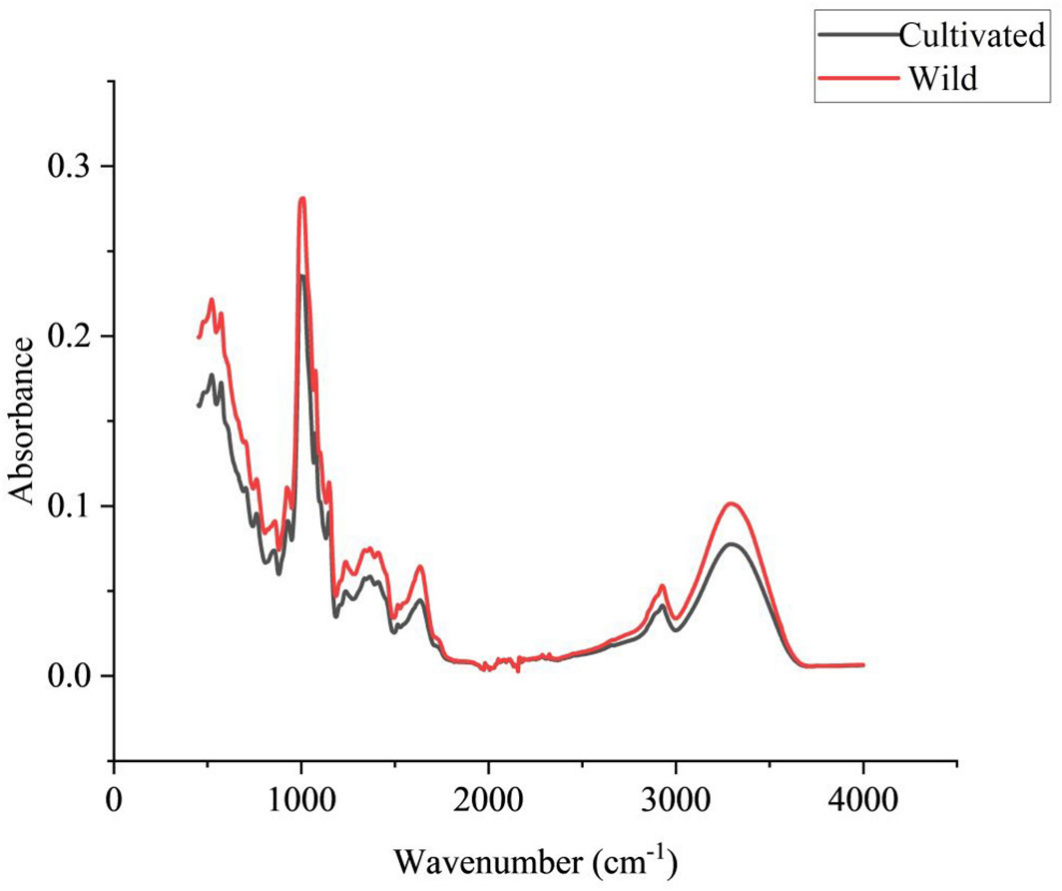
Raw mean spectral images of wild and cultivated *Gastrodia elata*.

The samples were scanned using an FT‐MIR spectrometer (PerkinElmer), and the instrument was equipped with a detector and a diamond single reflection universal attenuated total reflection (ATR) sampling accessory. The used software is SpectrumTM 10 ES. The spectra were acquired in the range 4000–450 cm^−1^ with a resolution of 4 cm^−1^. Each sample was scanned three times with each scan being 64 and the average was taken as the final spectrum. The original mean spectra of cultivated and wild *G. elata* are shown in Figure 2.

Spectroscopic instruments are subject to various interferences in the actual use process. The samples have different particle sizes and uneven distribution, as well as problems with light scattering and baseline instability (Robert & Gosselin, 2022). The result is that there is some redundant information or invalid information in the obtained spectral data. The absence of spectral data processing increases the time to train the model and reduces the model accuracy. Therefore, it is necessary to preprocess the spectral data before building the model. Yan et al. (2022) used a variety of preprocessing methods before building the model with the aim of minimizing such physical effects. The use of preprocessing methods can reduce the adverse effects of the physical properties of the sample on the spectroscopic measurement process. MSC and SNV can reduce the scattering effect of the spectra; the use of derivatives can separate overlapping peaks; SG can smooth out spurious peaks (Caused by noise; Kademi et al., 2019; Robert & Gosselin, 2022).

The Kennard Stone algorithm was used, and 70% of the total samples (wild: 44, cultivated: 76) were used as the training set and 30% (wild: 19, cultivated: 33) as the test set for subsequent SVM and PLS‐DA model building. Data preprocessing is performed by using 1D, 2D, MSC, SNV, and SG (third‐order derivatives with a window size of 15) and combinations of them.

### 2.3 Principal component analysis (PCA)

**FIGURE 3:**
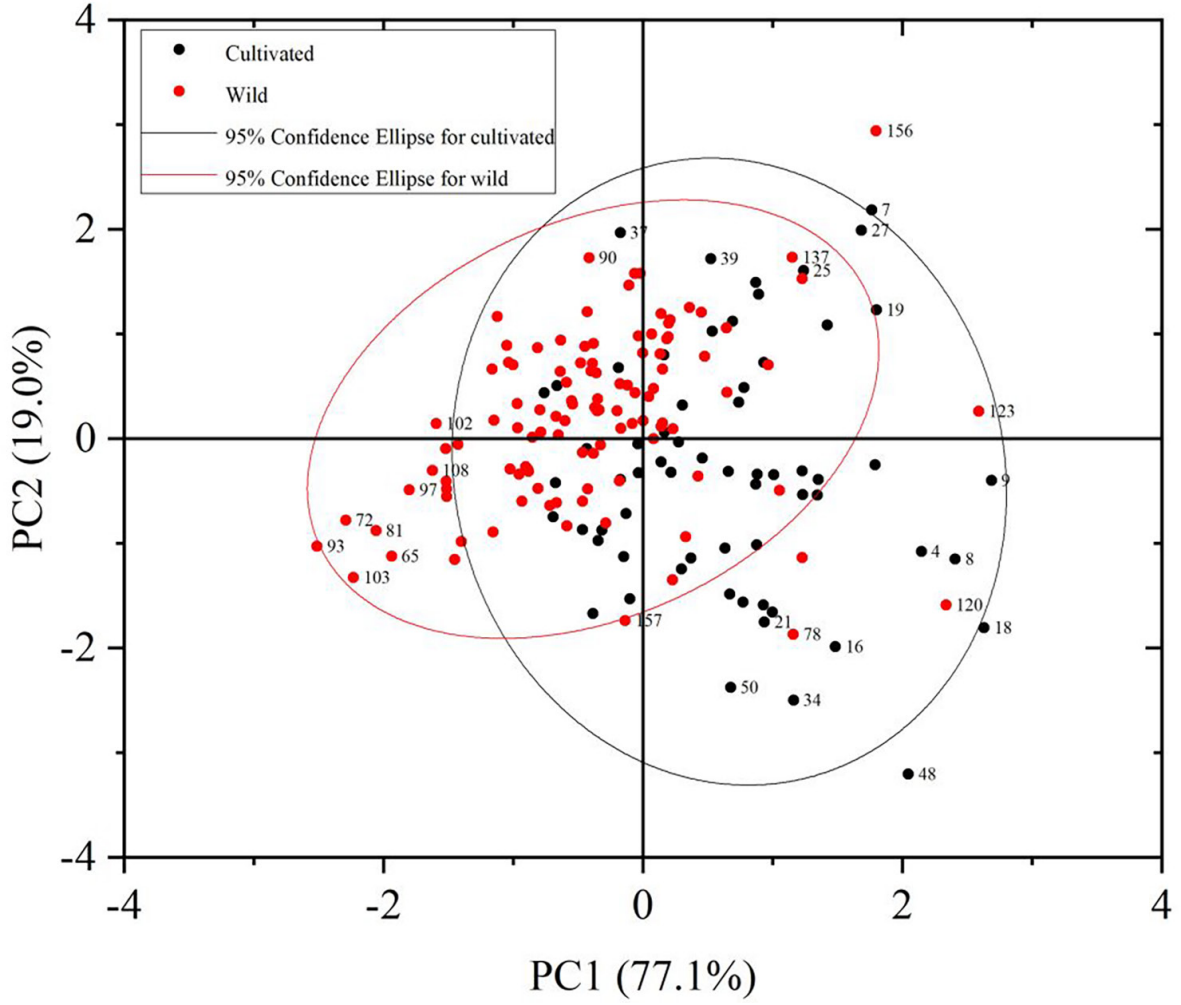
Principal component analysis score plots for wild and cultivated *Gastrodia elata*.

Principal component analysis, as an exploratory analysis technique, enables the classification of samples using information about their own characteristics. The visualization of the analysis results enables visualization of whether there are significant differences between the samples under study. For example, Da Costa Filho et al. (2022), in their study of adulteration of skim milk powder, used PCA to conclude that milk powder adulterated with lactose was similar to pure milk powder. Visualization of results was done by PCA, which can point the approach to subsequent experiments. In cases where it is difficult to differentiate between samples, researchers need to use supervised methods to solve the problem. The PCA score plots of cultivated and wild *G. elata* are shown in Figure 3.

### 2.4 PLS‐DA and SVM

PLS‐DA and SVM models, corresponding to linear and nonlinear problems, are frequently used in traditional chemometrics. PLS‐DA is capable of solving multicollinearity problems between variables. It is a supervised method to artificially classify and label the studied samples before model building. PLS‐DA is constructed by PLS combined with discriminant analysis, while partial least squares is an evolution of PLS regression algorithm with linear classification methods (Gautam et al., 2021). The building of model is based on an independent variable X matrix (spectral data) and a dependent variable Y matrix (classification labels; Wang et al., 2022). SVM is a nonlinear supervised method that has excellent performance in solving classification problems (Dankowska & Kowalewski, 2019; Teye et al., 2014). It demonstrates some advantages in solving nonlinear and high‐dimensional problems with small samples (Li et al., 2018).

These two models were built using the same preprocessing methods before comparing the performance of the models on this basis. The performance comparison of the two models is shown in Table 3. The test set confusion matrix plots of the PLS‐DA model are shown in Figure S1, and the training set hyperplane and test set classification results of the SVM model are shown in Figure S2.

### 2.5 Building of 3DCOS‐ResNet

**FIGURE 4:**
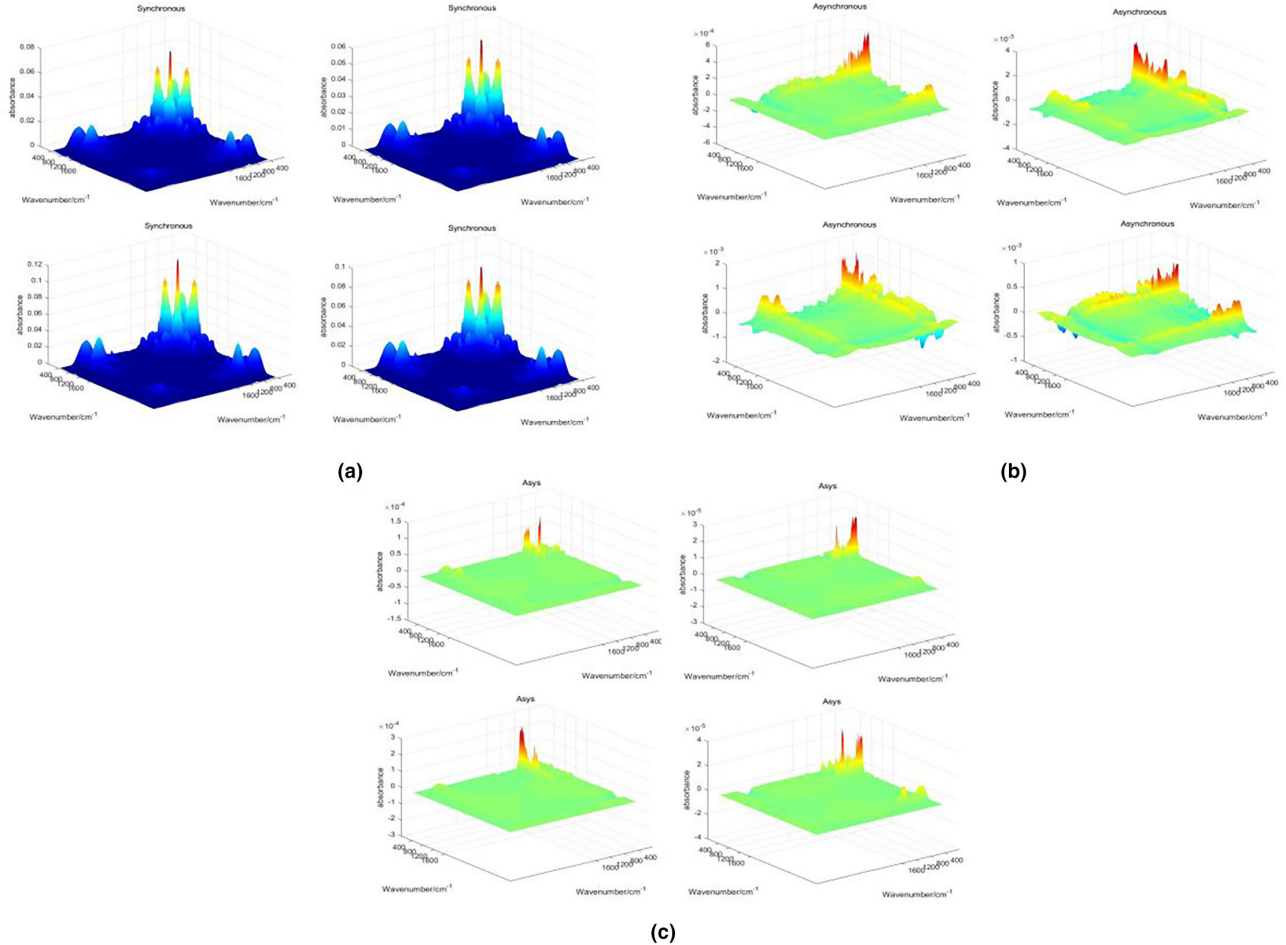
Three‐dimensional correlation spectral images (a, synchronous three‐dimensional spectral images; b, asynchronous three‐dimensional spectral images; c, integrated three‐dimensional spectral images).

The 3DCOS image categories generated using the software Matlab R2017a (Figure 1) are divided into synchronous, asynchronous, and integrated. There are 172 images in each category, for a total of 516 images. Eighty‐four images per category (172) were used as the training set, 36 as the test set, and the remaining 52 images were used as the external validation set. The generated images are saved in JPEG format for use in the 3DCOS‐ResNet model building (Figure 1). The spectral band of 4000–425 cm^−1^ utilized in this study was used for image generation, and secondly, the spectral data used before image generation were not preprocessed. The reason for this is that there is no change in the performance of the model under preprocessing (Yue et al., 2021). Yan et al. (2022) used the raw spectral data directly to build the model with 100% accuracy and a loss value of 0.041, which is not much different from that of the model built after the optimal preprocessing method. The 3DCOS images are shown in Figure 4.

(1) $$S\left(v\right)=\left\{\begin{array}{c} s\left(v,t1\right)\\ s\left(v,t2\right)\\ \ldots \\ s\left(v,\mathrm{tm}\right) \end{array}\right.$$

The spectral intensity is represented by the column vector *S*, the variables are represented by *V*, the perturbation time interval is represented by *t*, and m denotes the number of times (Noda, 2014).

(2) $$\Phi \;\left(v_{1},v_{2}\right)=\frac{1}{m-1}\;S{\left(v_{1}\right)}^{T}S\left(v_{2}\right)$$

(3) $$\Psi \;\left(v_{1},v_{2}\right)=\frac{1}{m-1}\;S{\left(v_{1}\right)}^{T}N\;S\left(v_{2}\right)$$

(4) $$I\;\left(v_{1},v_{2}\right)=\Phi \;\left(v_{1},v_{2}\right)\;\Psi \;\left(v_{1},v_{2}\right)=\frac{1}{{\left(m-1\right)}^{2}}\;\left[S{\left(v_{1}\right)}^{T}\;S\left(v_{2}\right)\right]\;\left[S{\left(v_{1}\right)}^{T}\;N\;S\left(v_{2}\right)\right]$$

where Φ, Ψ, and *I* are the expressions for the synchronous, asynchronous, and integrated three‐dimensional correlation strengths, respectively.

(5) $$\left\{\begin{array}{c} N_{\mathrm{jk}}=0，j=k\\ N_{\mathrm{jk}}=\frac{1}{\pi \left(k-j\right)},j\neq k \end{array}\right.$$

*N* is the expression in the case of equal or unequal *j* rows and *k* columns of the Hilber Noda matrix. The evaluations of the 3DCOS‐ResNet model are shown in Table 4. The building of the 3DCOS‐ResNet model was done by the software Matlab R2017a and the modeling method was referenced from Li et al. (2022).

### 2.6 Evaluation methods of the models

After the model has been built, it is particularly important to evaluate the performance of the model. The goodness or badness of a model cannot be judged by the accuracy rate of the training and testing sets alone. The evaluation of a model should be comprehensive and scientific, and cannot be evaluated by only one metric. Therefore, this study evaluates the PLS‐DA, SVM, and 3DCOS‐ResNet models with multiple metrics to comprehensively evaluate the overall effectiveness of the models.

The evaluation metrics of PLS‐DA model are *R* ^2^, *Q* ^2^, root mean square error of evaluation (RMSEE), root mean square error of cross‐validation (RMSECV), root mean square error of prediction (RMSEP), and accuracy of the model, respectively. The *R* ^2^ represents the fitting ability of the model and *Q* ^2^ represents the prediction ability of the model, and the closer the value is to 1, the better. The results of the model after 200 permutation tests are shown in Figure S3. The results of the evaluation metrics of the PLS‐DA model are shown in Table 1.

**TABLE 1:** The results of each evaluation metrics of PLS‐DA model.

Model	Preprocessing	Dataset	Number	Acc of train set (%)	Acc of test set (%)	RMSEE	RMSECV	RMSEP	*R* ^2^	*Q* ^2^
PLS‐DA	RAW	Train set Test set	Cultivated (76) Wild (44) Cultivated (33) Wild (19)	100	100	0.0766	0.1777	0.1251	0.9777	0.8918
	1D			100	100	0.1009	0.1788	0.1745	0.9591	0.8498
	**2D**			**100**	**100**	**0.0959**	**0.1669**	**0.1289**	**0.9624**	**0.9069**
	SNV			99.17	100	0.1725	0.3135	0.1638	0.8836	0.6046
	MSC			99.17	100	0.1828	0.3206	0.1778	0.8681	0.5854
	SG			100	100	0.1571	0.24703	0.1664	0.9035	0.7608
	SNV + 2D			100	100	0.1088	0.1829	0.1278	0.9511	0.8672
	MSC + 2D			100	100	0.1089	0.1829	0.1278	0.9511	0.8671
	SG + 2D			100	100	0.0931	0.1723	0.1489	0.9649	0.8904

Abbreviations: 1D, first‐order derivative; 2D, second‐order derivative; Acc, accuracy; MSC, multiplicative scattering correction; PLS‐DA, partial least squares discrimination analysis; RMSECV, root mean square error of cross‐validation; RMSEE, root mean square error of evaluation; RMSEP, root mean square error of prediction; SG, Savitzky–Golay; SNV, standard normal variate transformation.

The better model is indicated by the use of bolding.

The evaluation metrics of the SVM model are penalty parameter *c*, kernel parameter *g*, sensitivity (Sen), specificity (Spe), effectiveness (Eff), and accuracy (Acc). The values of parameters *c* and *g* are obtained by the grid search method. The optimal range of *c* is 2–2^−2^ and the range of *g* is 2 × 10^−4^ to 2 × 10^−2^. The choice of parameters *c* and *g* for the SVM model is particularly important because the values of *c* and *g* vary considerably during the model‐building process. Within a reasonable range, the larger the value of *c* is, the stronger the generalization ability of the model is; the larger the value of *g* is, the smaller the number of support vectors is. Therefore, the size of *g* value indirectly affects the training speed of the model. The results of the evaluation metrics of the SVM model are shown in Table 2.

**TABLE 2:** The results of each evaluation metrics of SVM model.

Model	Preprocessing	*c*	*g*	Acc of train set (120), %	Acc of test set (52), %
SVM	RAW	8.19 × 10^3^	6.10 × 10^−5^	98.33	63.46
	1D	1.81 × 10^2^	3.45 × 10^−4^	99.17	100
	**2D**	**2**	**7.81 × 10** ^**−3**^	**100**	**100**
	SNV	3.46 × 10^5^	8.76 × 10^−6^	97.5	63.46
	MSC	5.24 × 10^5^	2.70 × 10^−6^	98.33	63.46
	SG	8.19 × 10^3^	1.22 × 10^−4^	98.33	100
	**SNV + 2D**	**5.66**	**3.91 × 10** ^**−3**^	**100**	**100**
	**MSC + 2D**	**5.66**	**3.91 × 10** ^**−3**^	**100**	**100**
	**SG + 2D**	**2.83**	**7.81 × 10** ^**−3**^	**100**	**100**

Abbreviations: 1D, first‐order derivative; 2D, second‐order derivative; Acc, accuracy; *c*, penalty parameter; *g*, nuclear parameter; MSC, multiplicative scattering correction; SG, Savitzky–Golay; SNV, standard normal variate transformation; SVM, support vector machine.

The better models are indicated by the use of bolding.

The evaluation metrics of 3DCOS‐ResNet model are loss value, number of iterations (epochs), Acc, Sen, Spe, and Eff. The closer the loss value is to 0, the closer the other values are to 1 which indicates that the model works better.

(6) $$\mathrm{Acc}=\frac{\mathrm{TP}+\mathrm{TN}}{\mathrm{TP}+\mathrm{FP}+\mathrm{TN}+\mathrm{FN}}$$

(7) $$\mathrm{Sen}=\frac{\mathrm{TP}}{\mathrm{TP}+\mathrm{FN}}$$

(8) $$\mathrm{Spe}=\frac{\mathrm{TN}}{\mathrm{TN}+\mathrm{FP}}$$

(9) $$\mathrm{Eff}=\sqrt{\mathrm{Sen}\times \mathrm{Spe}}$$

where TP represents true‐positive samples; TN represents true‐negative samples; FP represents false‐positive samples; FN represents false‐negative samples. Sen is the ability of the model to identify samples from the target class, and Spe is the ability of the model to identify samples from the nontarget class. Equation 9 is the relationship equation between the two, representing the efficiency of the model (Oliveri & Downey, 2012).

### 2.7 Introduction of the used software

PCA and PLS‐DA model were built by the software SIMCA 14.1; SVM model was built by Matlab R2017a; 3DCOS images were generated by the software Matlab R2017a; and 3DCOS‐ResNet model was built by the software Spyder (Anaconda3).

## 3 RESULT AND DISCUSSION

### 3.1 Chemical composition analysis based on FT‐MIR spectral images

The raw average spectral images of wild and cultivated *G. elata* are shown in Figure 2. From the spectral images, the peak shapes of the two samples were basically the same, except for some differences in absorbance. From Figure 2, it was inferred that the content values of the chemical components were different between wild and cultivated *G. elata*. The specific wave number bands corresponding to chemical bonds need to be further analyzed. The C–O, C–C stretching, and C–O–H bending at 1014 cm^−1^ may belong to the vibrational band of C‐O on deoxyribose in DNA (Bureau et al., 2019). The absorption peak near 1220 cm^−1^ may be the phosphodiester functional group of DNA/RNA polysaccharide (Naumann, 2001). The absorption peak at 1240 cm^−1^ may be the ${\mathrm{PO}}_{2}^{-}$ of nucleic acids, phospholipids, and phosphorylated proteins, which was caused by the asymmetric stretching vibration of phosphodiester ${\mathrm{PO}}_{2}^{-}$ (Bellisola & Sorio, 2012). The complex absorption band corresponding to 1300–1500 cm^−1^ is mainly caused by lipids, CH_2_ and CH_3_ bending modes of proteins, and cyclic vibrations of nucleic acids (Naumann, 2001). The absorption peak at 1635 cm^−1^ is probably due to the C=O stretching of the amide I band (Bellisola & Sorio, 2012). 3000–2800 cm^−1^ may be the spectral band corresponding to C‐H of aliphatic or cyclic groups, and 2921 cm^−1^ contains aliphatic methylene bands, which are asymmetric stretches of CH_2_ groups (Ribeiro et al., 2001; Rosa et al., 2015; Sisouane et al., 2017). The chemical composition corresponding to the characteristic peak near 3276 cm^−1^ is the hydroxyl group and the N–H structure in the amide, and the absorption peak is related to the N–H and O–H stretching (Cao et al., 2022; Hell et al., 2016).

The entire spectral band was analyzed to decipher the specific chemical composition corresponding to the 4000–450 cm^−1^ range of wild and cultivated *G. elata*. Models were developed to distinguish between the two based on the differences in absorbance and characteristic fingerprint profiles.

### 3.2 PCA

As shown in Figure 3, PC1 and PC2 explained 77.1% and 19% of the total sample variance, respectively. Wild *G. elata* samples were mainly concentrated in the negative half‐axis of PC1, and cultivated *G. elata* were mainly concentrated in the positive half‐axis of PC1. However, there were still overlapping indistinguishable parts between the two samples, from which we can also conclude that there is some similarity between the two samples. This is reflected in Figure 2, where the spectral images show that the peak shapes of the two types of samples are similar, but there are differences in absorbance. Indirectly, the difference in the content of each chemical component between wild and cultivated *G. elata* was indicated, which was confirmed by Pei et al. (2022). PCA, as an unsupervised chemometric method, relies on the sample's own characteristics that make it difficult to fully cluster two classes of samples. This is also a step of exploratory analysis to help us provide ideas for subsequent problem solving. Therefore, we later used a supervised chemometric approach and deep learning to solve this problem.

### 3.3 Analysis and comparison of chemometric models

The PLS‐DA model was built on the basis of the datasets obtained under nine different preprocessing methods, and the results are shown in Table 1. The results of the PLS‐DA model under the nine pretreatments after 200 permutation tests are shown in Figure S3, and none of the models showed overfitting (the left‐hand side measurements are all lower than the right‐hand side true values, and the *y*‐values of *Q* ^2^ are all less than zero). The PLS‐DA model built under 2D gave the best results, and the model under MSC gave the worst results, followed by SNV. The model built based on the raw spectral data works satisfactorily instead, better than any other preprocessing methods except 2D. The reason may be that due to the relatively obvious differences existing between wild and cultivated *G. elata*, a satisfactory result can be achieved by using a supervised linear analysis technique. In the choice of preprocessing methods, except for 2D, none of the preprocessing methods could reduce the three root mean square error values of the model, nor could they improve the *R* ^2^ and *Q* ^2^ of the model.

The SVM models were built in the same process as PLS‐DA, all under nine different datasets. The results of the models are shown in Table 2, and the best results are finally obtained under 2D, MSC + 2D, and SG + 2D. The parameters *c* and *g* of the model are within a reasonable range. Although the *c* and *g* of the model built by the SNV + 2D method were within a reasonable range, the accuracy of the test set was only 63.46%. The *c* and *g* values of the model built under other preprocessing are not within a reasonable range. There is a risk that the model is overfitted or that too many support vectors reduce the model training speed.

Table 3 shows the comparison results of PLS‐DA model and SVM model. Taking the best modeling results of SVM as a reference, only three methods, 2D, MSC + 2D, and SG + 2D, are selected for comparison. Because the model effects of SNV + 2D and MSC + 2D are the same, only one of them is selected for comparison. The Sen, Spe, Acc, and Eff of both models reach 100%. And the combined comparison of Tables 1, 2, 3 concludes that the PLS‐DA model gives better results. The accuracy of the test set is 100% regardless of the preprocessing method or the original data. Except for SNV and MSC, the model has good results for all the metrics under the other methods.

**TABLE 3:** The results of comparing each evaluation metrics of PLS‐DA and SVM models.

Models	Preprocessing	Sen	Spe	Eff	Acc
PLS‐DA	2D	1	1	1	1
	MSC + 2D	1	1	1	1
	SG + 2D	1	1	1	1
SVM	2D	1	1	1	1
	MSC + 2D	1	1	1	1
	SG + 2D	1	1	1	1

Abbreviations: 2D, second‐order derivative; Acc, accuracy; Eff, effectivity; MSC, multiplicative scattering correction; PLS‐DA, partial least squares discrimination analysis; Sen, sensitivity; SG, Savitzky–Golay; Spe, specificity; SVM, support vector machine.

### 3.4 Deep learning model

**FIGURE 5:**
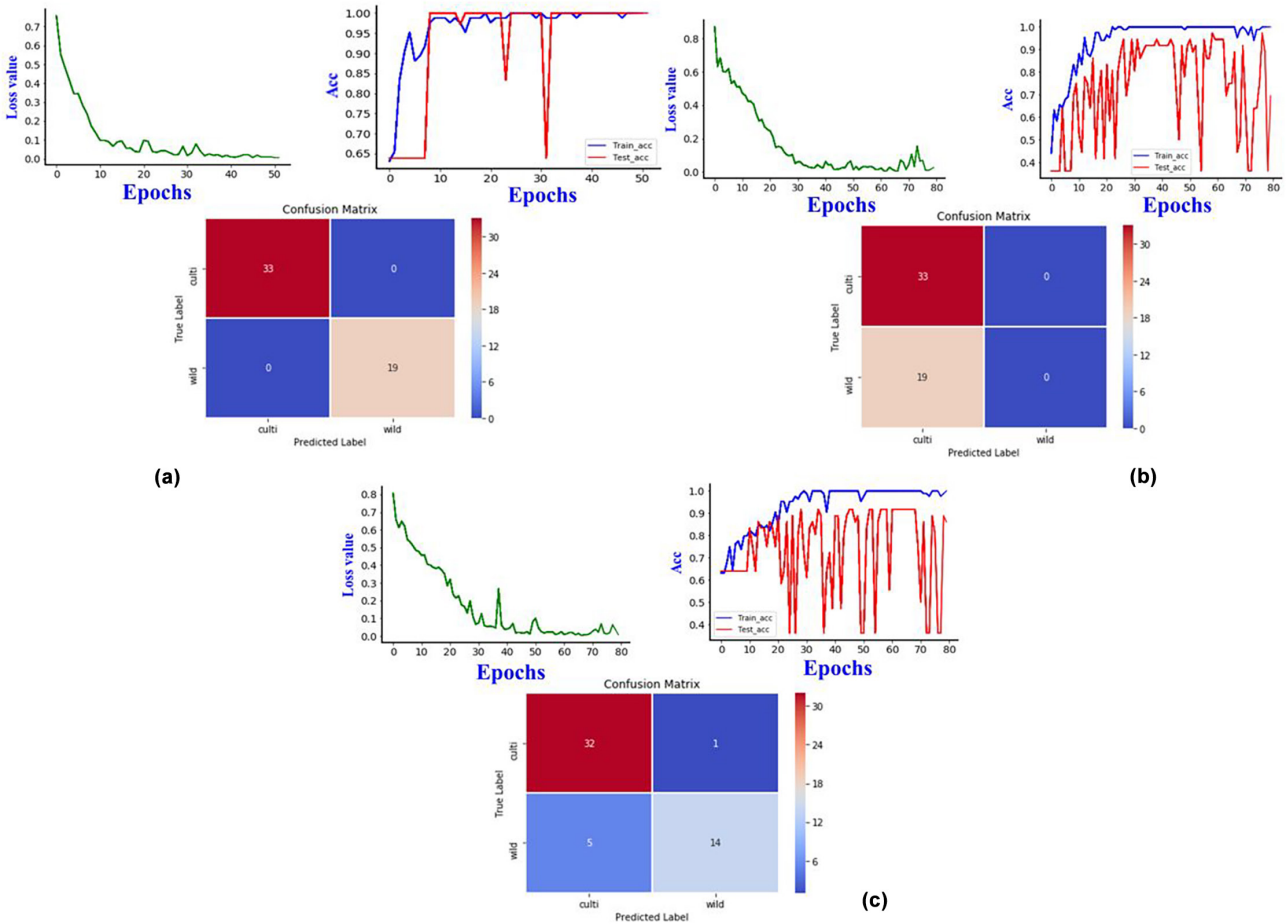
Plots of results for the training set, test set, loss values, and external validation set for the three models (a, synchronous model; b, asynchronous model; c, integrated model).

The evaluation results of the three 3DCOS‐ResNet models are shown in Table 4. In terms of the accuracy of the external validation set of the models, the synchronous‐3DCOS‐ResNet model gives the best results followed by the integrated and asynchronous models. The loss values, accuracy, and external validation set results for the synchronous, asynchronous, and integrated models are shown in Figure 5. When building the synchronous model, the performance of the model is unstable until the number of iterations is 32, and the test set accuracy fluctuates. After the number of iterations exceeds 35, the model's training set and test set accuracies stabilize at 100%. To stabilize the model performance, the epoch is finally set to 46. The model works better with 100% accuracy by external validation test. The asynchronous and integrated models were not satisfactory, and the test set accuracy still did not trend to a stable value when the number of iterations reached 79. During this process, the accuracy of the training set was able to stabilize at 100%, but the accuracy of the test set fluctuated widely.

**TABLE 4:** Comparison of 3DCOS‐ResNet models.

Model	Classifications	Epochs	Acc of train set (%)	Acc of test set (%)	Acc of EV (%)	Loss value	Sen	Spe	Eff
3DCOS‐ResNet	Synchronous	46	100	100	100	0.005	1	1	1
	Asynchronous	79	100	61	63.46	0.027	63.46%	63.46%	63.46%
	Integrated	79	100	86	88.46	0.007	88.46%	88.46%	88.46%

Abbreviations: 3DCOS‐ResNet, three‐dimensional correlated spectral images combined with residual convolutional neural networks; Acc, accuracy; Eff, effectivity; EV, external verification; Sen, sensitivity; Spe, specificity.

A comprehensive comparison shows that the synchronous‐3DCOS‐ResNet model works best. Firstly, it builds the model based on the original spectral data compared to PLS‐DA and SVM and does not require preprocessing of the data to achieve a satisfactory result. This operation can significantly save time by skipping the step of processing data and proceeding directly to modeling. 3DCOS‐ResNet models are faster to identify compared to traditional chemometric models as concluded by Yue et al. (2021) and Yan et al. (2022); secondly, the loss value, number of iterations, accuracy, Sen, Spe, and Eff of the model are at a satisfactory level. The 3DCOS‐ResNet model was established for the first time for the identification of wild and cultivated *G. elata*. The performance of the model was evaluated by various metrics, and the preliminary judgment was that the model can play a fast and accurate identification effect.

## 4 CONCLUSION

In this study, 3DCOS combined with a deep learning model was applied for the first time to the identification of wild and cultivated *G. elata*, and a comparison of synchronous, asynchronous, and integrated models yielded the best results for the synchronous‐3DCOS‐ResNet model. All evaluation metrics of the synchronous model are satisfactory, while the performance of the asynchronous and integrated models is unstable, and the accuracy of the test set fluctuates to a large extent that is difficult to stabilize. Before the establishment of PLS‐DA and SVM models, the spectral data need to be preprocessed, and the evaluation metrics of the models can be improved under the appropriate preprocessing methods. However, the advantages are not significant compared with the 3DCOS‐ResNet model. The synchronous‐3DCOS‐ResNet model achieves a stable level of accuracy for the training and test sets at a lower number of iterations (46), and 100% accuracy for the external validation set. Mao et al. (2023) used Backed‐propagation (BP) neural network technology to solve the shelf‐life prediction problem for channel catfish fillets, and the number of iterations of the model was over 8700 to achieve such results. At the same time, no preprocessing of spectral data is required for building deep‐learning models. This saves a lot of time cost for studies with large samples and makes the advantages of the model more obvious. Thus, the overall results of the synchronous‐3DCOS‐ResNet model are satisfactory as concluded from the results of this paper. It was able to solve the identification problem of wild and cultivated *G. elata*, but the ability of the asynchronous and integrated deep learning models could be improved. In addition, the sample size in this study was not very large, and this reason may have contributed to the large difference in the effectiveness of the synchronous, asynchronous, and integrated models. Further experiments will be conducted to optimize the performance of the models by expanding the sample size as much as possible in future studies.

## AUTHOR CONTRIBUTIONS

**Shuai Liu:** Writing – original draft (equal). **Honggao Liu:** Software (equal); supervision (equal). **Jieqing Li:** Methodology (equal); visualization (equal). **Yuanzhong Wang:** Formal analysis (equal); resources (equal).

## CONFLICT OF INTEREST STATEMENT

There is no conflict of interest between the authors throughout this study.

## Supporting information


Data S1

## Data Availability

The data used in the article are confidential.
