# Supplementary material for: Building deep learning and traditional chemometric models based on Fourier transform mid‐infrared spectroscopy: Identification of wild and cultivated Gastrodia elata
Source: Food Sci Nutr. 2023 Jul 11;11(10):6249–59. doi: 10.1002/fsn3.3565 (PMC10563693; doi:10.1002/fsn3.3565)
Supplement: Supplementary file 1 — Data S1 [file FSN3-11-6249-s001.docx]

Table S1 Detail information of samples.

Table S1 Detail information of samples.

| Samples | Classification | Number | Origin |
| --- | --- | --- | --- |
| *Gastrodia elata* | Cultivated | 51 | Deqin County, Diqing Prefecture, Yunnan Province |
|  |  | 58 | Yiliang County, Zhaotong City, Yunnan Province |
|  | Wild | 63 | Dongshan Town, Qilin District, Qujing City, Yunnan Province |

Figure S1. Confusion matrix plots for PLS-DA models with 9 data sets.

Figure S2. Plots of training set hyperplane and test set classification results for SVM models with 9 data sets (A: RAW; B: first-order derivative (1D) ; C: second-order derivative (2D); D: standard normal variate transformation (SNV); E: multiplicative scattering correction (MSC); F: Savitzky-Golay; G: SNV+2D; H: MSC+2D; I: SG+2D).

Figure S3. Plots of R^2^ and Q^2^ relationships for the PLS-DA model after 200 permutation tests.


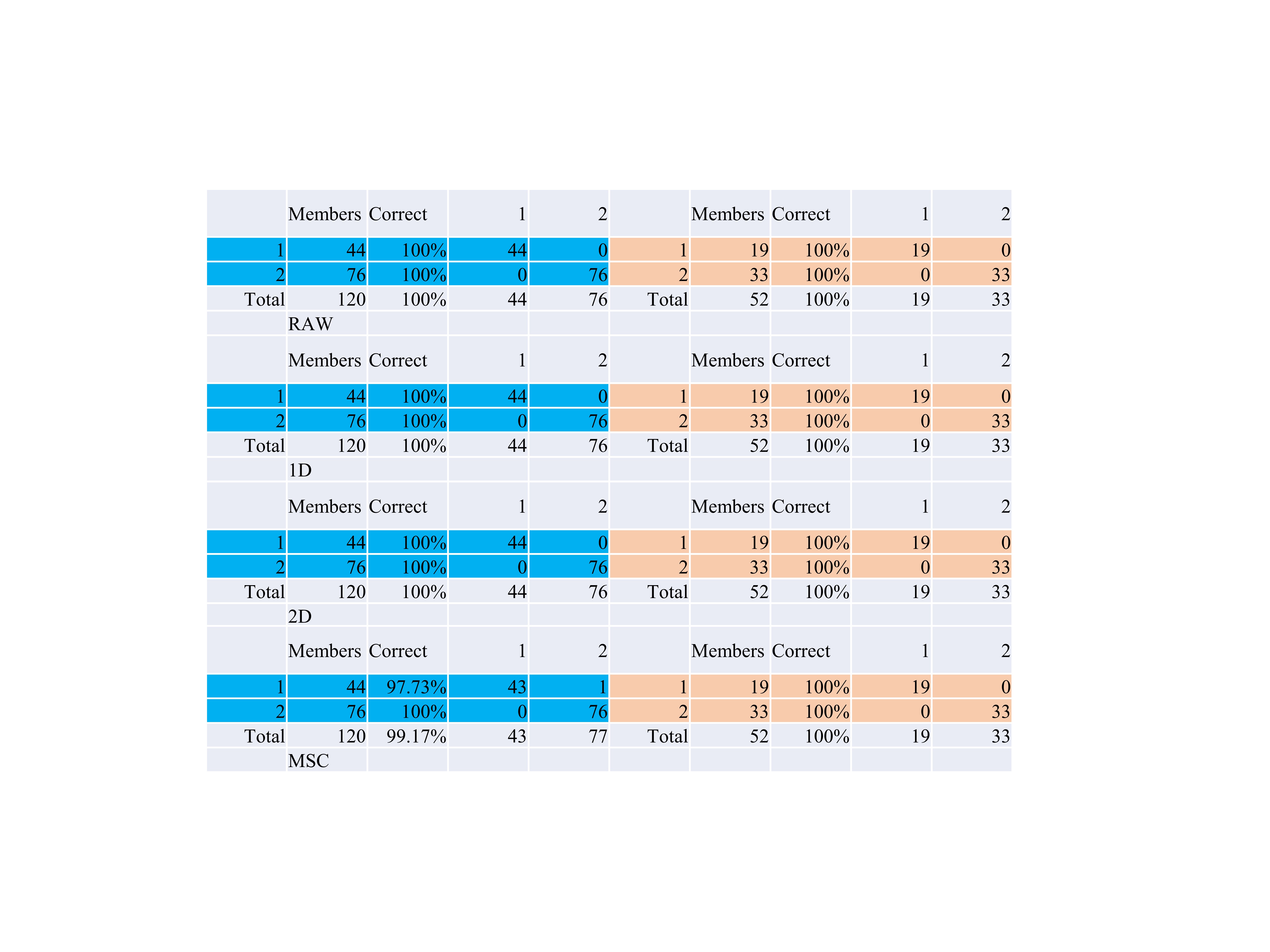


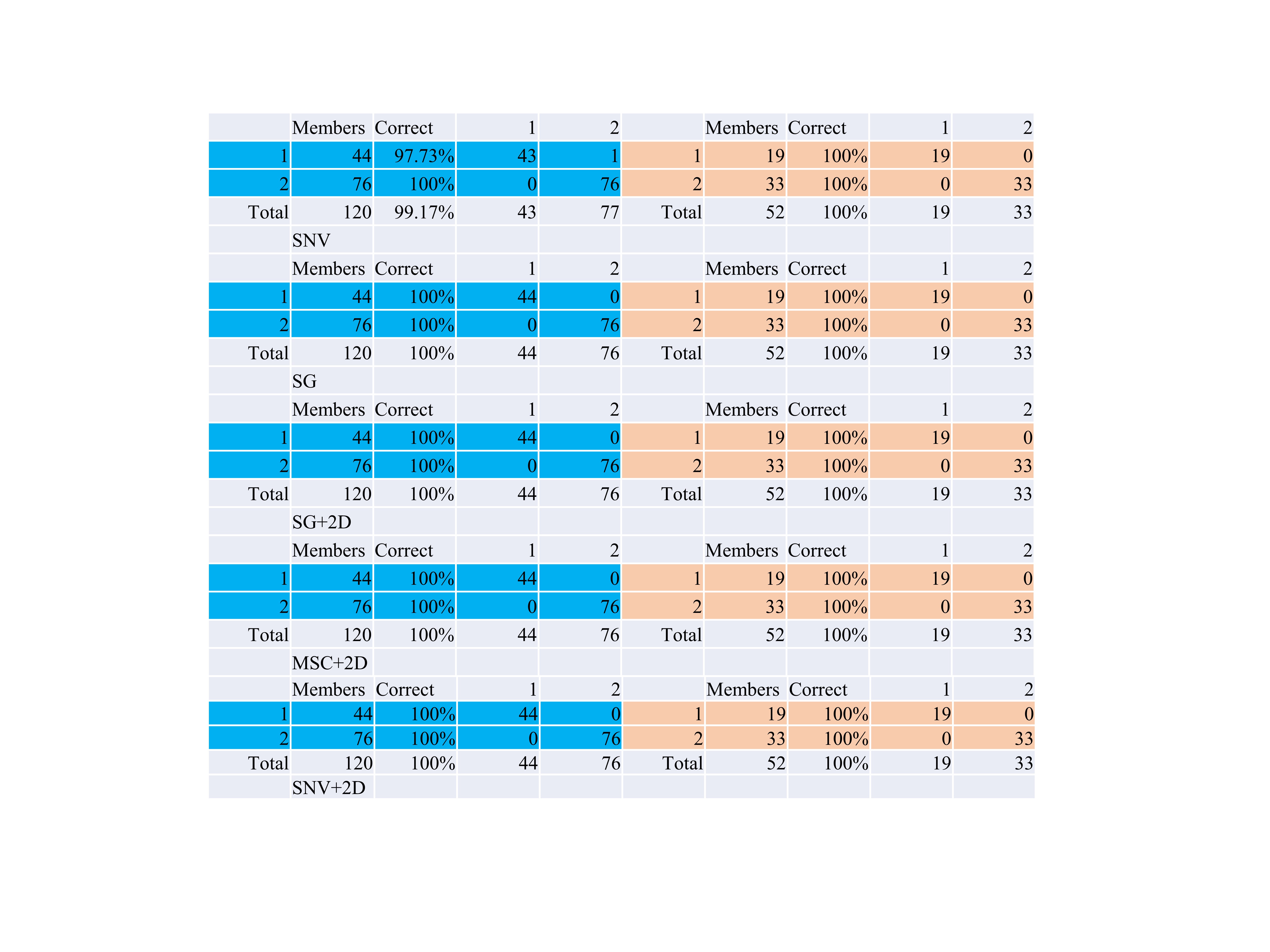


Figure S1. Confusion matrix plots for PLS-DA models with 9 data sets.


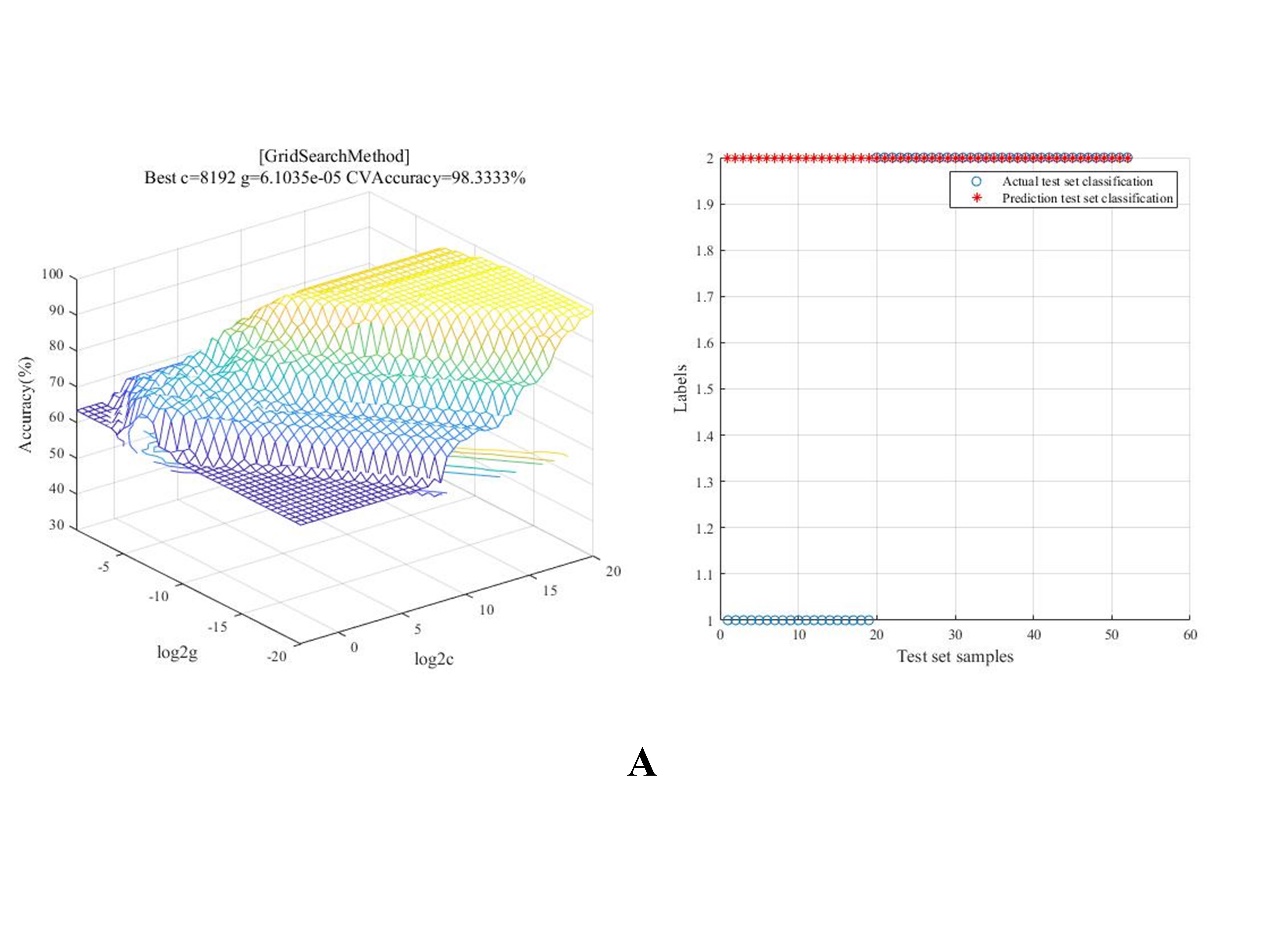


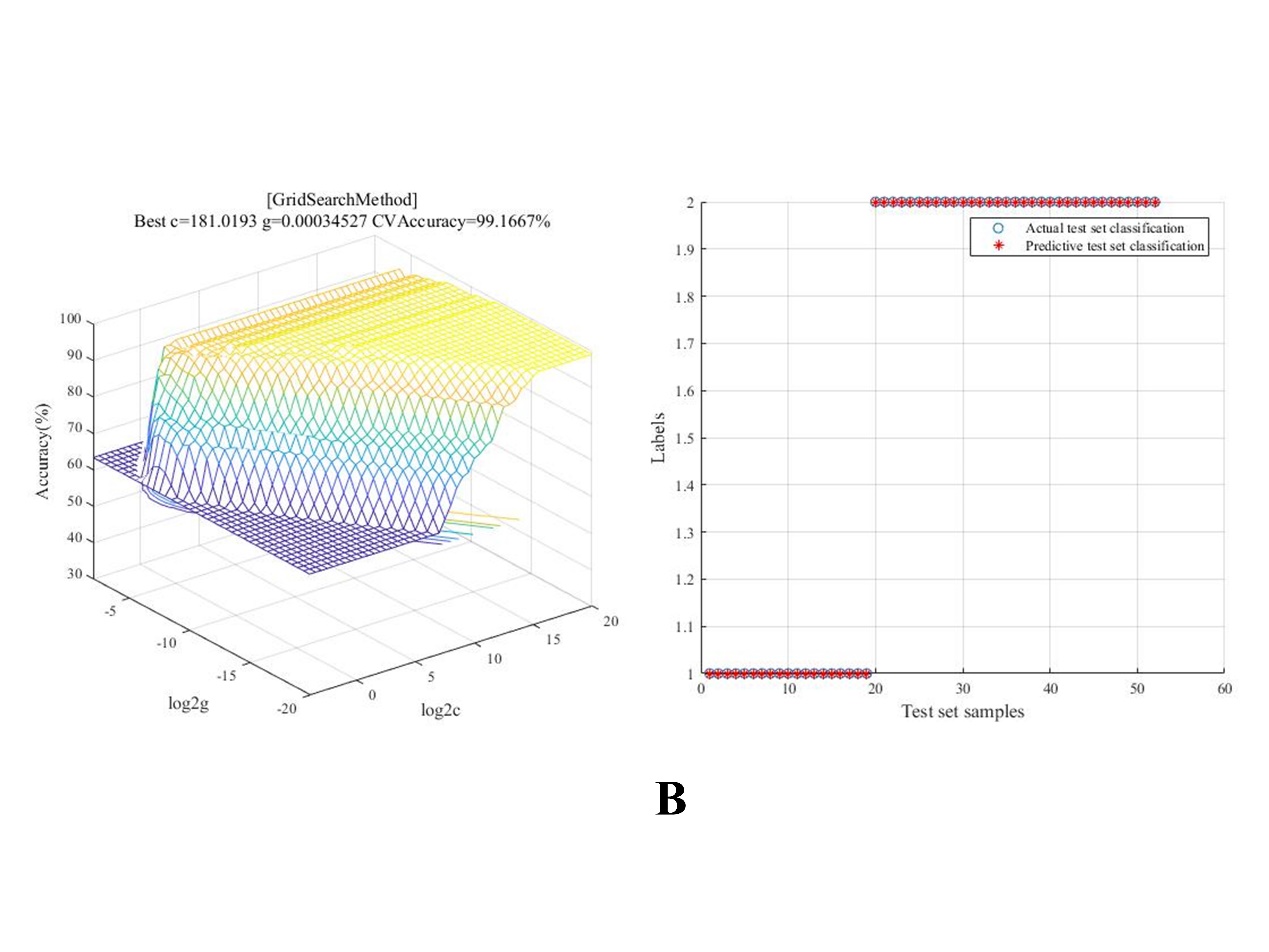


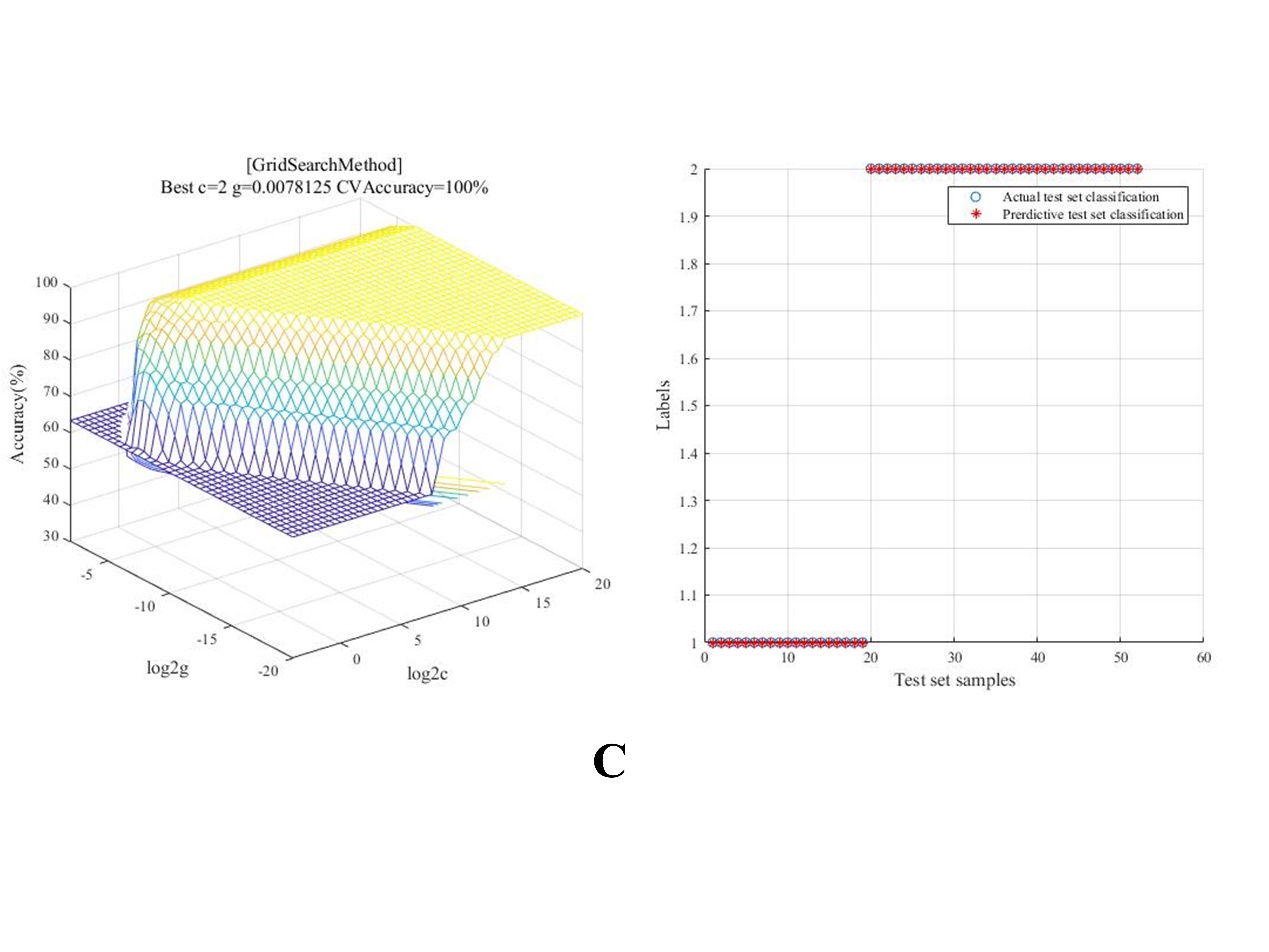


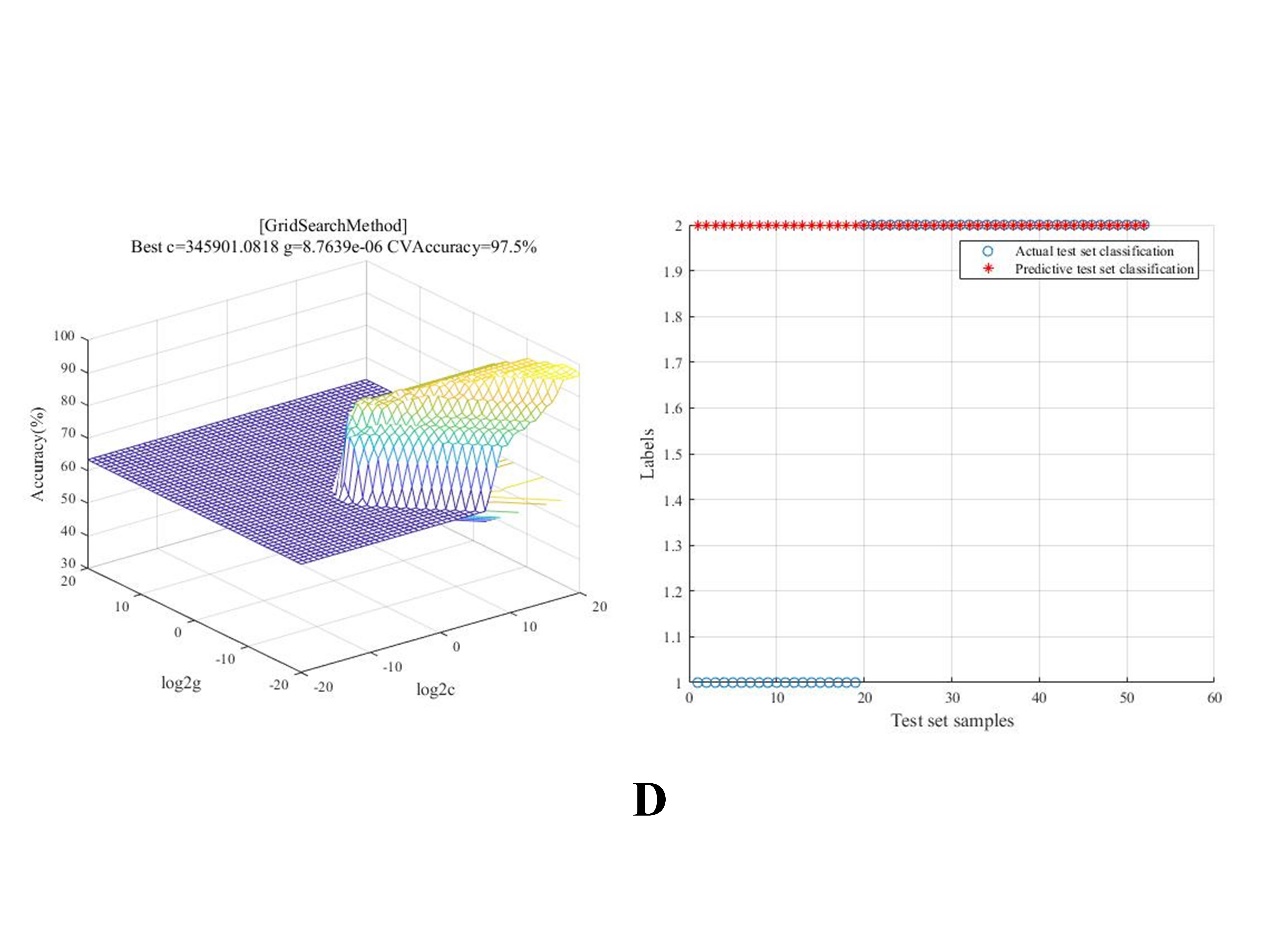


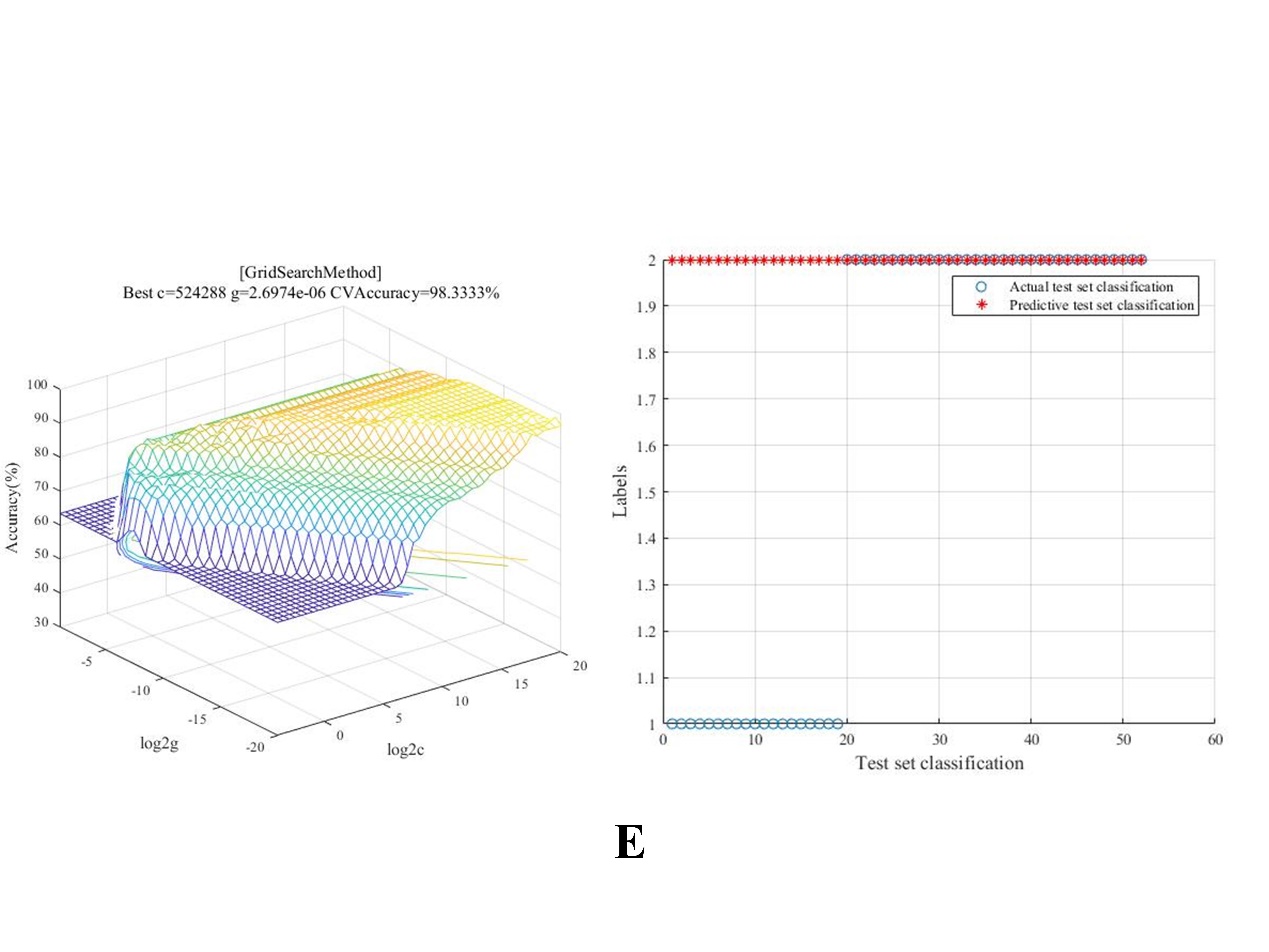


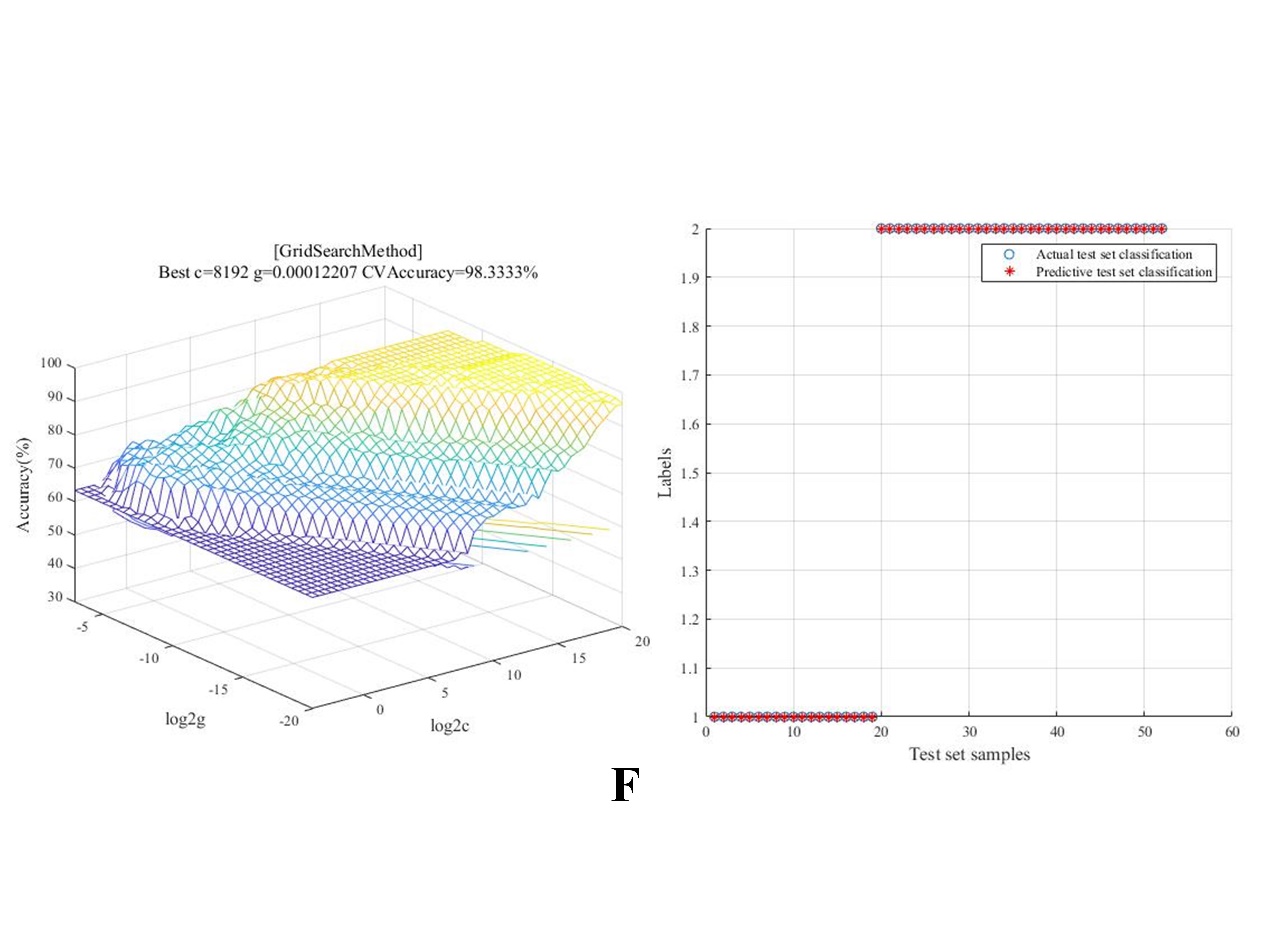


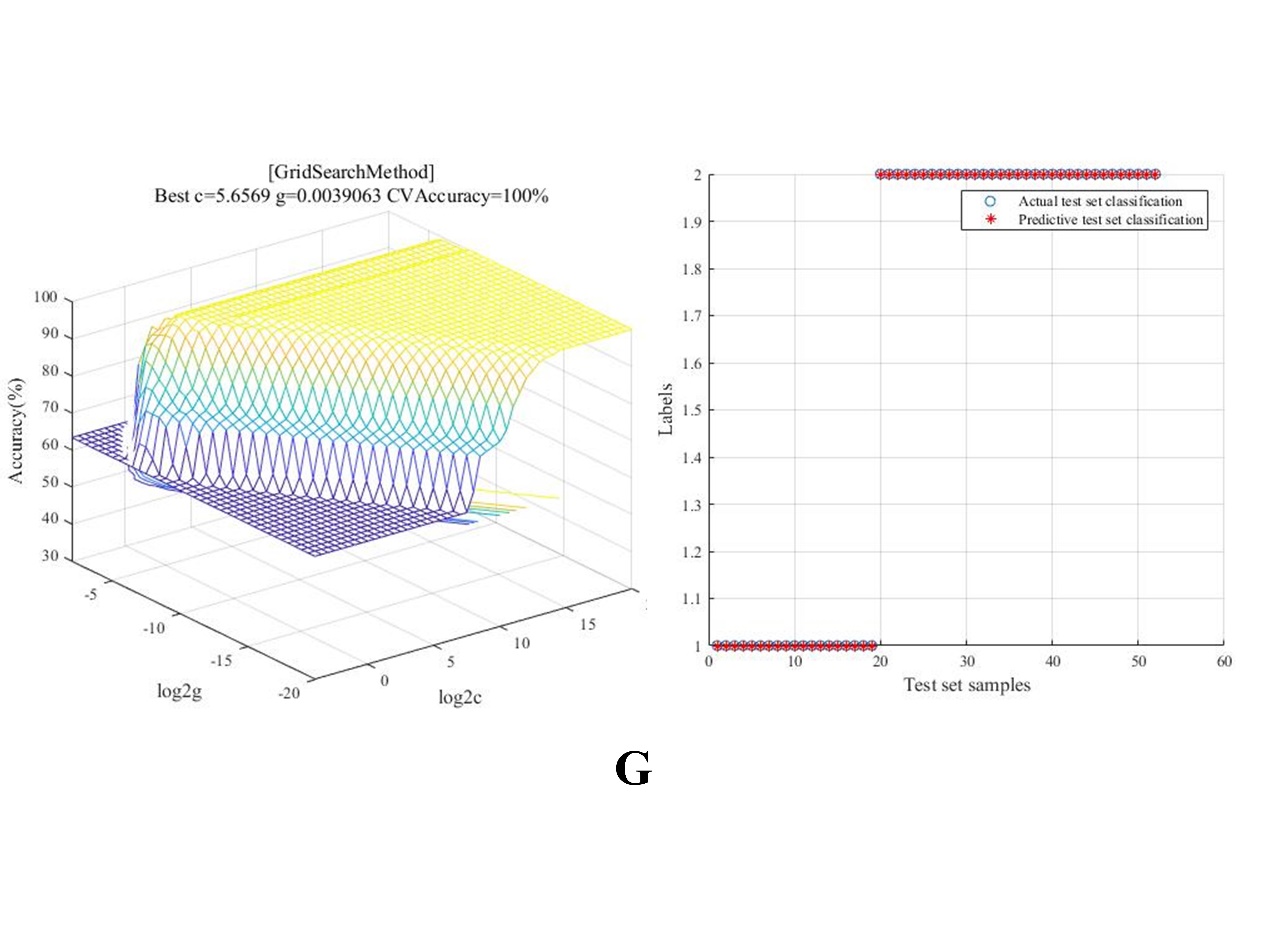


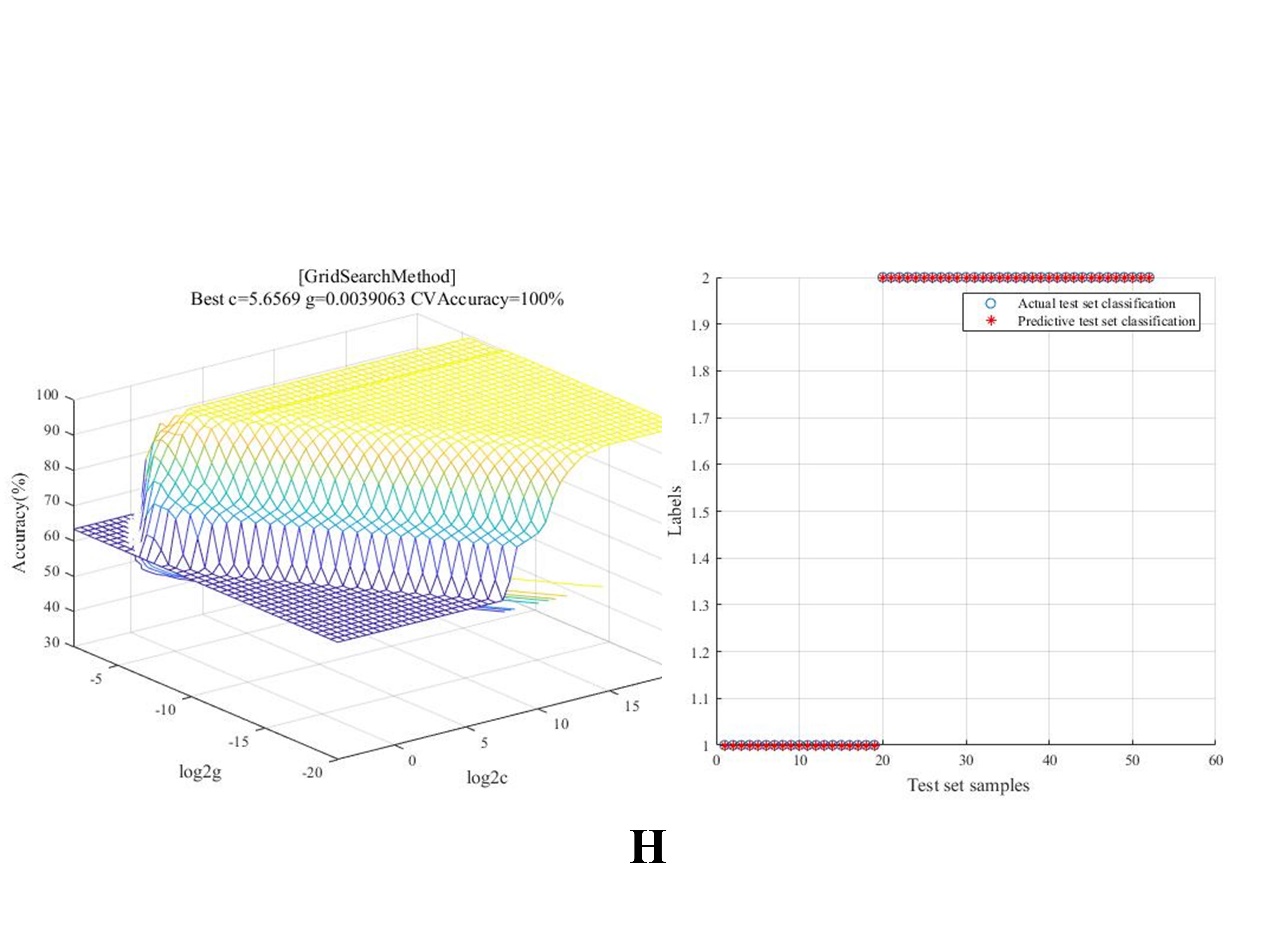


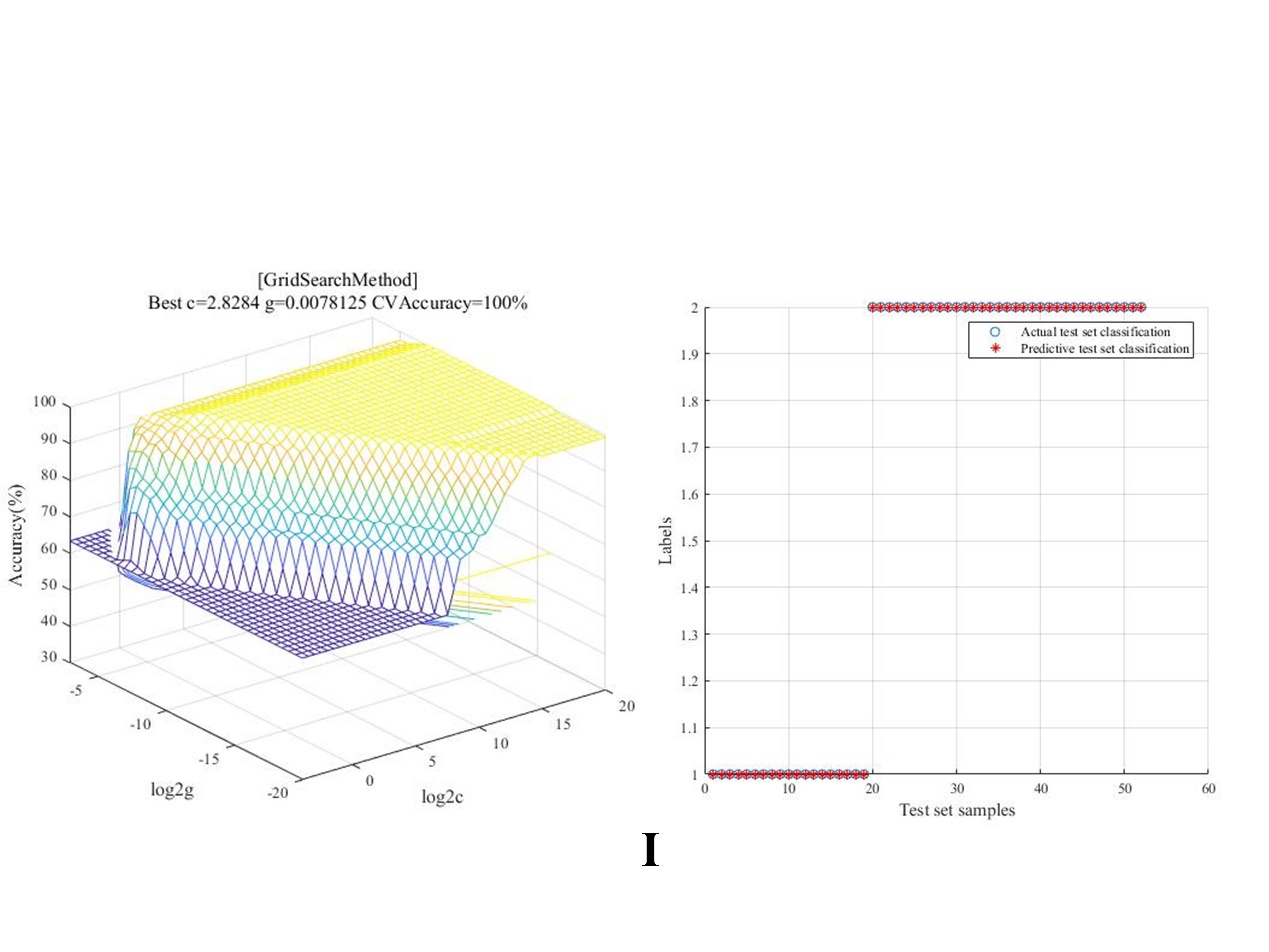


Figure S2. Plots of training set hyperplane and test set classification results for SVM models with 9 data sets (A: RAW; B: first-order derivative (1D) ; C: second-order derivative (2D); D: standard normal variate transformation (SNV); E: multiplicative scattering correction (MSC); F: Savitzky-Golay; G: SNV+2D; H: MSC+2D; I: SG+2D).


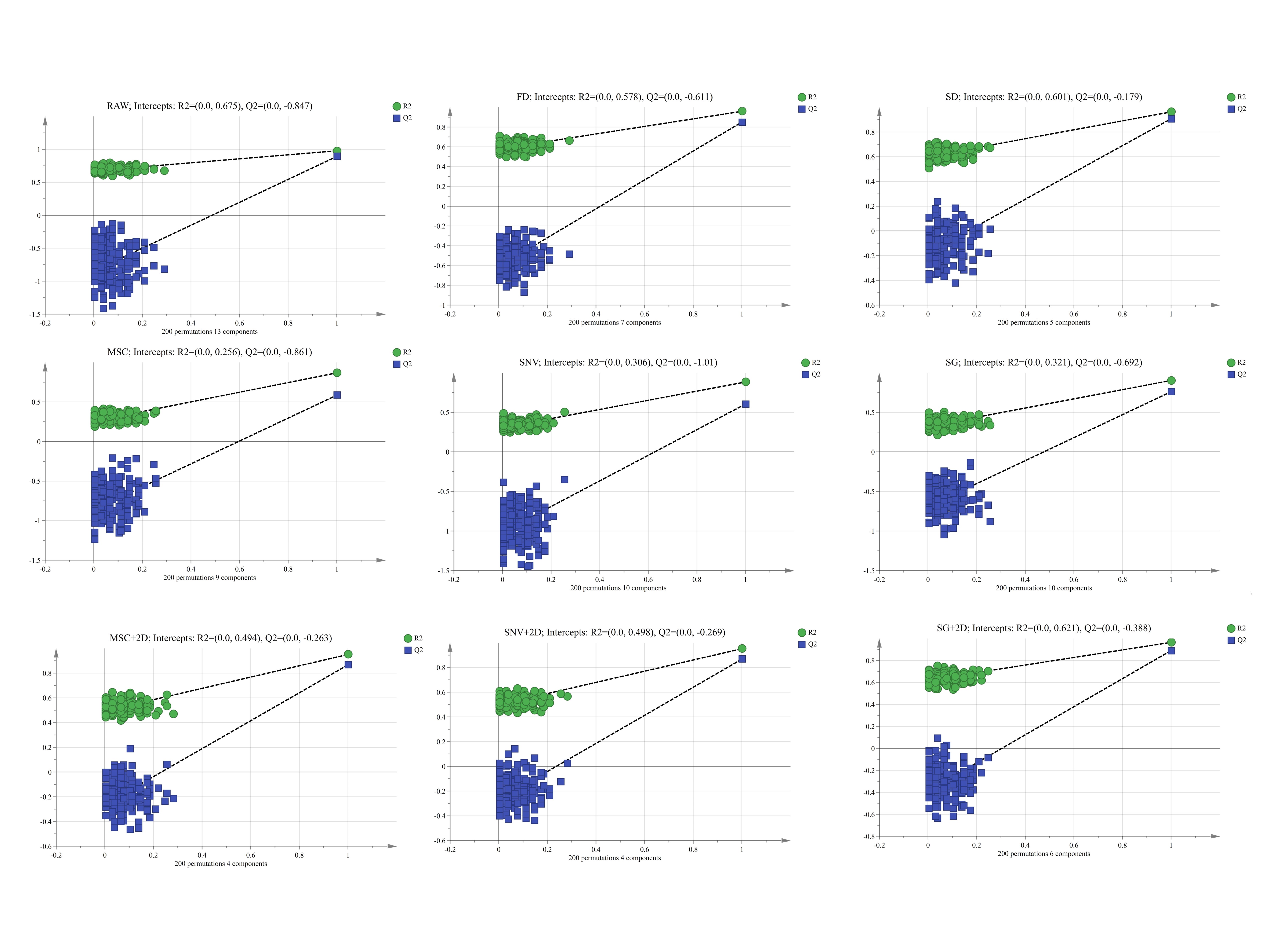


Figure S3. Plots of R^2^ and Q^2^ relationships for the PLS-DA model after 200 permutation tests
